# An Unusual Erupted Complex Composite Odontoma: A Rare Case

**DOI:** 10.1155/2013/106019

**Published:** 2013-01-03

**Authors:** Dawasaz Ali Azhar, Mohammad Zahir Kota, Sherif El-Nagdy

**Affiliations:** ^1^Department of Oral Medicine and Radiology, College of Dentistry, King Khalid University, Abha, Saudi Arabia; ^2^Department of Oral Surgery, College of Dentistry, King Khalid University, Abha, Saudi Arabia; ^3^Department of Oral Pathology, Faculty of Dentistry, Mansoura University, Dakahliya, Egypt

## Abstract

Odontomas are malformations of the dental tissues and may interfere with the eruption of the associated tooth. Complex composite odontoma (CO) was described as a distinct entity for the first time by Broca in 1866. This lesion takes place due to the developmental disturbances where the dental components are laid down in a disorganized manner, due to failure of normal morphodifferentiation. Very few cases of erupted complex composite odontomas have been reported in the literature. The case reported here is of an odontoma found in the left mandibular body, associated with an impacted second molar of a 17-year-old Saudi male. Under local anesthesia the odontoma was surgically removed. Histopathological examination confirmed the diagnosis of CO. The impacted second molar which was left in the mandibular body erupted clinically after 6 months. Erupted CO is rarely seen in the mandibular left body. The early diagnosis, followed by a proper treatment at the right time, will result in a favorable prognosis.

## 1. Introduction

According to the latest classification of the World Health Organization (WHO, 2005), two types of odontomas can be found: complex odontomas and compound odontomas, the latter being twice as common as the former. Odontomas erupting into the oral cavity are rare. The first case was published in 1980, and since then only 17 cases have been reported in the literature. Eight of the 17 cases were complex composite odontomas (COs); the rest were compound odontomas. Pain, swelling, and infection were the most common symptoms, and 13 cases presented an impacted tooth associated with it [1]. COs are found in the mandibular posterior region over impacted teeth which can reach up to several centimeters in size. Radiographically, these lesions manifest as a radiopaque solid mass with occasional nodular elements and are surrounded by a fine radiolucent zone separated from the normal bone by a well-defined cortication line [2].

## 2. Case Description

**Figure 1:**
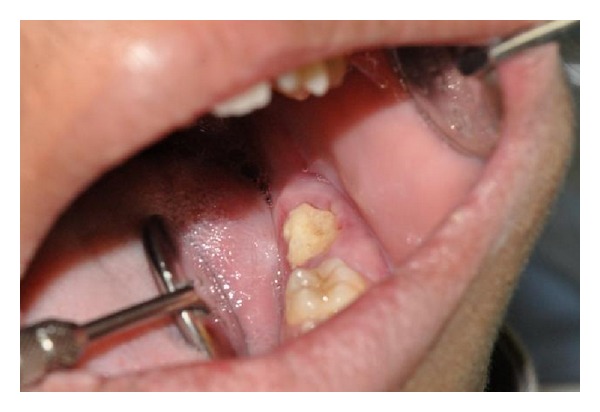
Intraoral preoperative photograph.

**Figure 2:**
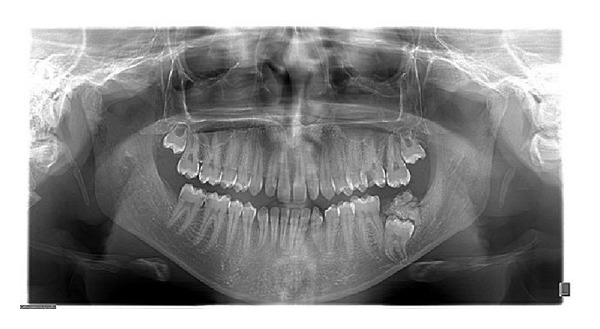
Orthopantomograph showing radiopaque structure with impacted mandibular left second molar.

**Figure 3:**
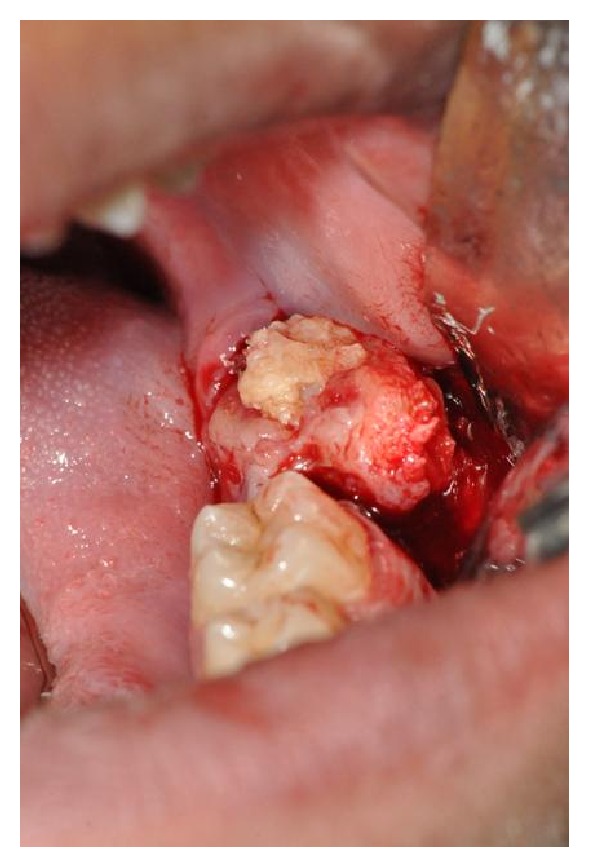
Intraoperative photograph showing excision in total.

**Figure 4:**
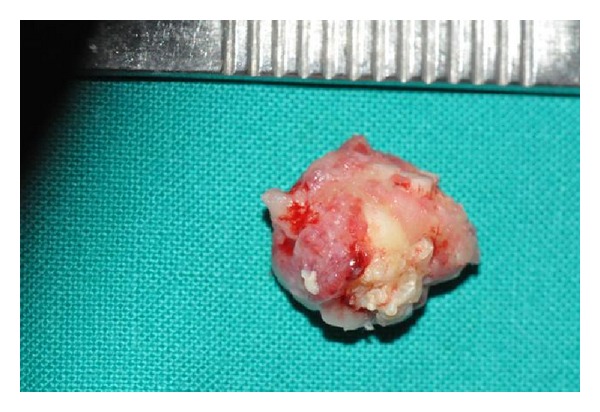
Excised specimen.

**Figure 5:**
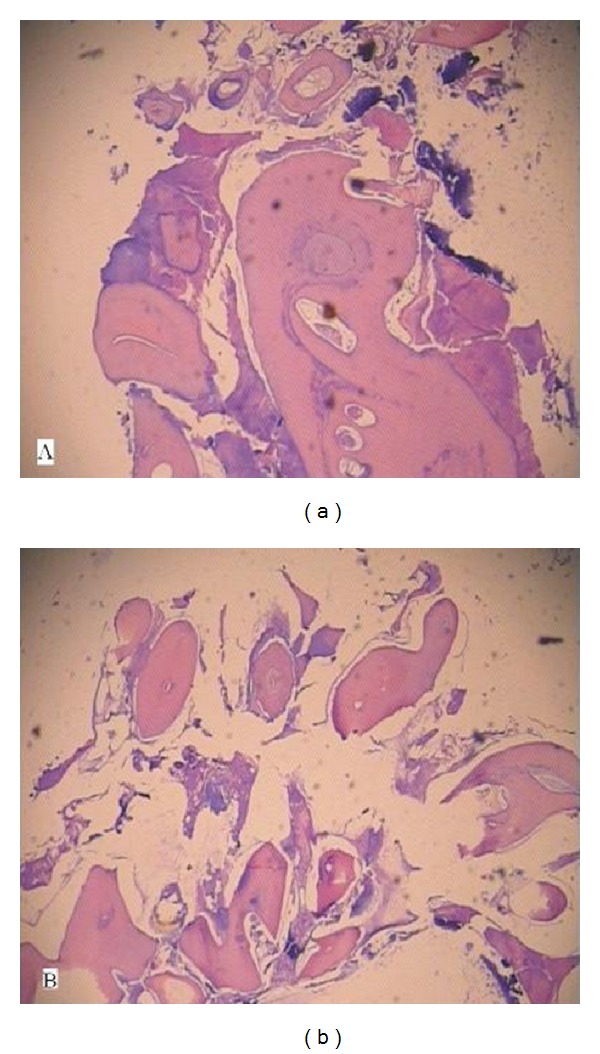
H&E stained decalcified section showing (a) disorganized dental tissue formed of irregular dentine masses containing multiple hollow circular spaces with pulp tissue and enamel matrix and (b) small areas of organized dental tissue resembling tooth structure with proliferating odontogenic epithelium seen in a scanty stroma.

**Figure 6:**
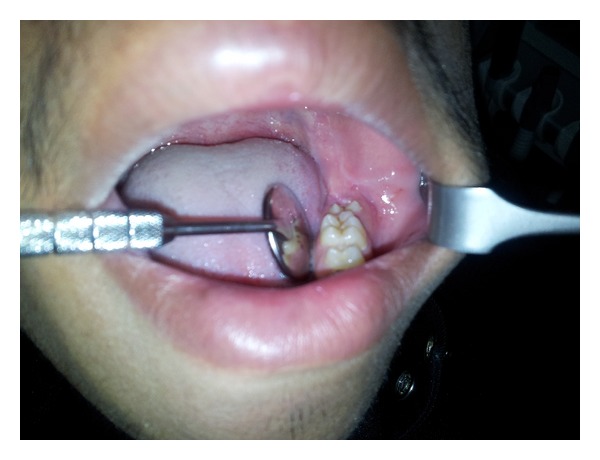
Postoperative clinical photograph (6-month followup) showing erupted mandibular left second molar.

**Figure 7:**
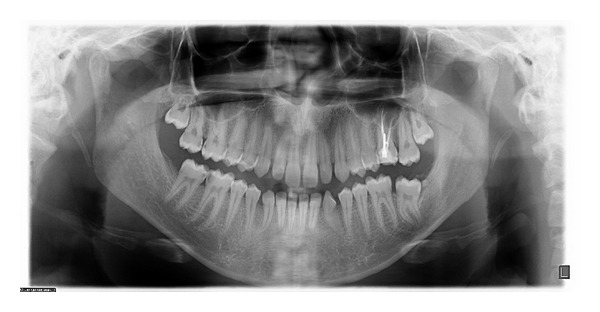
Orthopantomograph (6-month followup).

A 17-year-old male visited the dental OPD of King Khalid University, Abha, KSA, with a chief complaint of pain in upper left first molar tooth. Routine clinical examination revealed a partially erupted hard mass on the alveolar ridge distal to left mandibular first molar and missing mandibular second molar (Figure 1). Orthopantomograph revealed a unilateral solid single triangular radiopaque structure with apex towards the alveolar ridge (Figure 2). The radiopaque structure was irregular and had a radiolucent zone surrounding it. There was a presence of an impacted second molar with the root apices close to the left lower border of the mandible. Differential diagnoses made based on the clinical and radiological features included complex odontoma, cementoblastoma, ameloblastic fibroodontoma, and Pindborg's tumor. In our case there was a presence of the radiolucent rim around the lesion with the presence of dense radiopacity produced by enamel which helped to distinguish the odontoma. The above lesion was surgically excised under local anesthesia (Figures 3 and 4) and edges of the bones were rounded and the wound was rinsed with saline and sutured with Coated Vicryl 3/0 (Ethicon, Inc., Johnson and Johnson Company, USA). The second molar was left in situ for expected passive eruption. The postoperative course was uneventful. The excised specimen was sent for histopathological examination. The decalcified section showed disorganized dental tissue formed of irregular dentine masses containing multiple hollow circular spaces with pulp tissue and enamel matrix (Figure 5(a)). Other small areas of organized dental tissue resembling normal tooth structure are also seen (Figure 5(b)). Proliferating odontogenic epithelium is also seen in a scanty stroma. The above findings are consistent with complex composite odontoma. At the followup after 6 months it was observed clinically and radiographically (Figures 6 and 7) that the second molar had erupted in its usual position distal to lower left first molar.

## 3. Discussion

The WHO classification defines CO as follows: “a malformation in which all the dental tissues are represented, individual tissues being mainly well formed but occurring in a more or less disorderly pattern” [2]. The etiology of COs is unknown. Several theories have been proposed, including local trauma, infection, family history, and genetic mutation. It has also been suggested that odontomas are inherited from a mutant gene or interference, possibly postnatally with the genetic control of tooth development [3]. The relative frequency of CO among odontogenic tumors varies between 5% and 30% which means that this lesion is one of the most commonest odontogenic lesion/malformation. The majority of cases (83.9%) occur before the age of 30 with a peak in the second decade of life. The male : female ratio varies between 1.5 : 1 and 1.6 : 1 [4]. It is of interest to note that the majority of odontomas in anterior segment of jaw are compound composite in type (61%) whereas the majority in posterior segment are CO. Interestingly, both type of odontomas occurred more frequently on the right side of jaw than on the left, such presentation was not seen in our case [5]. Clinically CO is a painless, slow-growing, and expanding lesion that is usually discovered on routine radiographs of the jaw bones, or the failed eruption of a permanent tooth which may lead to the diagnosis of this lesion [3]. The first case of an erupted odontoma was described in 1980 by Rumel et al. The mean patient age was 25–35 years, thus confirming potential presentation of these lesions between the second and third decades of life [6]. The mechanism of odontoma eruption appears to be different from tooth eruption because of the lack of periodontal ligament and root in odontoma. Therefore the force required to move the odontoma is not linked to the contractility of the fibroblasts, as in the case for teeth. Although there is no root formation in odontoma, its increasing size may lead to the sequestration of the overlying bone and hence occlusal movement or eruption. An increase in the size of the odontoma over time produces a force sufficient to cause bone resorption [1].

Radiologically, the compound odontoma appears as a collection of tooth-like structures while composite type appears as a calcified mass with a radiodensity similar to tooth structure; both are further surrounded by a narrow radiolucent zone. However there was absence of any corticated border in our case as normally published in previous literature. Unerupted teeth are more commonly associated with compound composite odontoma. However, in our case, presence of unerupted tooth with CO was seen [5]. The lesion appears as a more or less amorphous, solitary mass of calcified material. In some cases, the lesion shows a radiating structure [7]. 

Rarely odontomas may form peripheral or soft tissue lesions in which they arise outside alveolar bone and may exfoliate or erupt. Such a rare erupted odontoma was present in our case [8].

Microscopically, this lesion consists primarily of a well-delineated, roughly spherical mass of a haphazard conglomerate of mature hard dental tissues. Some examples may include better-ordered, tooth-like structures [9]. Clear spaces and clefts that probably contain mature enamel lost in the process of decalcification are often seen. In some sections at the periphery of the mass, islands of pulp tissue in association with cords and buds of odontogenic epithelium can be found. However, the usual high degree of differentiation of the dental tissues reflects the late stage of morphodifferentiation and maturation of odontogenesis. A thin, fibrous capsule and, in some cases, a cyst wall is seen surrounding the lesion [4].

Conservative surgical enucleation is considered to be the treatment of choice in most cases of CO. During the removal of pathological structures in the mandibular retromolar region in an early age, care should be taken to preserve bone structures on the anterior border of the mandibular ramus, because it might impair jaw development. If needed, a control X-ray should be taken during the surgery. The prognosis is always good since these tumors do not tend to recur [10]. As odontomas are often associated with impacted teeth, the possibility that eruption of the impacted tooth after a presumed obstructive odontoma has been surgically removed is an important issue [11]. In our case, taking into consideration the position of the associated impacted tooth and its presumable path of eruption, it was decided to leave it in situ for passive eruption. 

## 4. Conclusion

Odontomas rarely erupt into the mouth and tend to be associated with impacted teeth. Despite their benign nature, however, their eruption into the oral cavity can give rise to pain, inflammation and infection and different clinical appearance. The treatment of choice is surgical removal of the odontoma, followed by histopathological analysis. In the case of odontomas associated with impacted teeth, the latter should be preserved in situ when a favorable path of eruption exists for facilitating passive eruption of the impacted teeth into the oral cavity.
